# *Bach2*-deficient mice are prone to autoimmune pancreatitis but protected from high-fat diet-induced fatty liver disease

**DOI:** 10.3389/fimmu.2025.1639622

**Published:** 2025-10-15

**Authors:** Luise Ehlers, Anika Kasprick, Ottavia Agrifoglio, Natalie Gross, Nancy Ernst, Alanis Barbosa Gulde, Colin Osterloh, Noa Linn Brauckmann, Nicole Y. Fischer, Wendy Bergmann-Ewert, Ralf J. Ludwig, Katja Bieber, Robert Jaster

**Affiliations:** ^1^ Department of Internal Medicine, Division of Gastroenterology, Hepatology and Nutritional Medicine, Rostock University Medical Center, Rostock, Germany; ^2^ Lübeck Institute of Experimental Dermatology and Center for Research on Inflammation of the Skin, University of Lübeck, Lübeck, Germany; ^3^ Core Facility for Cell Sorting and Cell Analysis, Rostock University Medical Center, Rostock, Germany

**Keywords:** *Bach2*, mouse model, autoimmune pancreatitis, fatty liver disease, high-fat diet

## Abstract

**Background:**

Autoimmune pancreatitis (AIP) is a multifactorial disease caused by both genetic and environmental factors. Previous studies have implicated Bach2, a key regulator of adaptive immunity, in the pathogenesis of this disease. However, direct experimental evidence is lacking. Here, we used C57BL/6N mice with a targeted deletion of *Bach2* (*Bach2* knockout mice) to study their susceptibility to AIP under homeostatic conditions and in response to two AIP triggers: a high-fat diet (HFD) and polyinosinic:polycytidylic acid (poly I:C).

**Methods:**

In this multicenter preclinical study, *Bach2* wild type and knockout mice were maintained under homeostatic conditions, challenged with a HFD for 3 months, or treated with poly I:C for 6 weeks. The pancreata were examined histopathologically. Additionally, RNA sequencing and PamGene multiplex kinase activation measurements were performed. To assess the effects of the HFD, the livers were evaluated for the presence of fatty liver disease.

**Results:**

Consistent with the results of previous studies, *Bach2* knockout mice showed reduced growth and developed eosinophilic crystalline pneumonia, necessitating humane euthanasia at the age of 18 weeks. At 8 and 18 weeks of age, pancreatic infiltrates with lymphocytes typical of AIP were frequently detected in *Bach2* knockout mice but not in wild type animals without additional manipulations. RNA sequencing analyses and kinase activity assays revealed the activation of processes linked to adaptive immunity in the pancreatic tissues of *Bach2* knockout mice. Wild type mice treated with poly I:C showed lymphocytic infiltrates similar to those of untreated knockout mice, whereas HFD did not induce AIP. In *Bach2* knockout mice, HFD and poly I:C did not further enhance the disease. As expected, HFD-fed wild type mice developed fatty liver disease. Strikingly, the livers of *Bach2* knockout mice were almost free of fat and histological changes, such as hepatocyte ballooning and degeneration. The data obtained from the two project sites were highly consistent, indicating strong intersite reproducibility.

**Conclusion:**

*Bach2*-deficient C57BL/6N mice were prone to spontaneous AIP development. This could be due to disturbed immune homeostasis with dysregulated activation of adaptive immune system cells. The protective effect of *Bach2* deficiency against the development of fatty liver disease warrants further investigation.

## 1 Introduction

Autoimmune pancreatitis (AIP) is a rare but clinically important special form of chronic pancreatitis (CP). On one hand, it can be difficult to accurately differentiate AIP from pancreatic carcinoma, while on the other hand, unlike CP of other causes, it usually responds well to immunosuppressive therapy with steroids. The two best-established AIP subtypes are often associated with diseases in other organs. Subtype 1 or lymphoplasmacytic-sclerosing AIP, is considered a pancreatic manifestation of an IgG4 systemic disease, whereas subtype 2, characterized by granulocytic epithelial lesions, is often associated with ulcerative colitis (1, 2). Autoimmune diseases, including AIP, are multifactorial diseases influenced by interactions between environmental factors, such as changes in the microbiome and diet, and genetic factors (3–5). In AIP, both aspects are only partially understood. Considering that current therapies are largely symptomatic, have limited effectiveness, and are associated with numerous side effects, more specific and mechanism-based treatments are required. In this context, it is important to identify modifiable risk factors, among which environmental factors play a central role.

The genes associated with human AIP include the HLA alleles *DRB1^∗^16* and *HLA-DQB1^∗^05* (6), *FCRL3* (7), *CTLA-4* (8, 9), *KCNA3* (10), and specific *PRSS1* mutations (*PRSS1_IVS 2 + 56_60 delCCCAG* and *PRSS1_p.Leu81Met*) that were suggested to cause ectopic trypsinogen activation (11, 12). In previous studies, we used the MRL/MpJ mouse strain, a model of spontaneous AIP that can be further aggravated by injecting synthetic nucleic acids, polyinosinic: polycytidylic acid (poly I:C) (13, 14), to elucidate the genetic basis of the disease. By fine-mapping the quantitative trait loci (QTLs) of murine AIP, we identified two new putative risk genes for the disease in mice (4, 15): mitogen-activated protein kinase 7 (*Map3k7*) and *Bach2*. While the latter gene was the focus of this study, we postponed follow-up studies on MAP3K7 for the time being, as we did not observe any effect of the specific MAP3K7 inhibitor, takinib, on experimental AIP in a recent investigation (16).

The B lymphoid transcription repressor, BTB and CNC homology (BACH) proteins, including BACH1 and BACH2, belong to the family of cap ‘n’ collar and basic leucine zipper (bZip) transcription factors and are highly conserved in vertebrates. BACH proteins can form heterodimers with small musculoaponeurotic fibrosarcoma (MAF) and BACH2 also with basic leucine zipper ATF-like transcription factor (BATF) proteins via their bZIP domain (17, 18). The widely expressed BACH1 protein competes with nuclear factor erythroid 2-like 2 (NRF2) to bind MAFs, thereby blocking the transcription of heme oxygenase−1 (*HO-1*/*HMOX1*) and other oxidative stress response genes (19). BACH1 activation appears to play an unfavorable role in several chronic diseases. These include cancer (in which BACH1 promotes tumor cell proliferation and metastasis), neurodegenerative diseases, chronic inflammatory bowel diseases, pulmonary fibrosis, and skin diseases (17). BACH2 expression is largely restricted to the cells of the innate and adaptive immune systems and neuronal cells. The transcription repressor plays pivotal roles throughout hematological development and differentiation, specifically lineage commitment and the development of both innate and adaptive immune cells (reviewed in (18)). BACH2 controls the expression of an entire cluster of genes with essential functions in T cell activation, including those encoding many cytokines and cytokine receptors, via stretching or super-enhancers (20). *BACH2* haploinsufficiency can lead to a syndrome of *BACH2*-associated immunodeficiency and autoimmune disorders (21). Furthermore, polymorphisms in human gene loci are associated with multiple autoimmune diseases, such as type 1 diabetes (22), Crohn’s disease (23), celiac disease (24), and multiple sclerosis (25). The immunological phenotypes that are influenced by *BACH2* include blood lymphocyte, monocyte, and neutrophil counts (26) and IgG glycosylation (27). Murine *Bach2* is also considered a critical regulator of CD4^+^ memory T cell development (28), which, according to our previous studies, may be involved in the pathogenesis of experimental AIP (29). In agreement with these findings from preclinical model systems, Sasikala et al. observed reduced expression of *BACH2* in pancreatic tissues of CP patients and identified a *BACH2* gene variant (rs9111 in 5’-UTR) that was associated with advanced disease (30).

Overall, previous studies have suggested a possible role of *BACH2* in the pathogenesis of AIP. To investigate this role experimentally, we used mice with a targeted *Bach2* deletion in this study and examined their susceptibility to AIP under normal conditions and in response to two potential environmental triggers, poly I:C and a high-fat diet (HFD), which have been previously studied in MRL/Mp and outbred mice prone to disease development (4, 14, 31).

Consistent with our hypothesis, mice lacking *Bach2* developed AIP. Unexpectedly, a second phenotype was observed in these mice, which were protected from HFD-induced fatty liver disease. Considering the rapid increase in the prevalence of metabolic dysfunction-associated steatotic liver disease (MASLD, previously known as nonalcoholic fatty liver disease (NAFLD)) in humans (32), this novel aspect of *Bach2* function deserves further attention.

## 2 Materials and methods

### 2.1 Animals

C57BL/6N-Bach2^tm1b(EUCOMM)Wtsi^/leg mice (EMMA ID EM:09104) with Cre-mediated excision of the parental *Bach2^tm1a(EUCOMM)Wtsi^* allele were kindly provided as heterozygous individuals by the Helmholtz Zentrum Muenchen–German Research Center for Environmental Health (GmbH) and INFRAFRONTIER/The European Mouse Mutant Archive (EMMA) (33, 34). The mice were maintained as independent colonies in the local animal facilities of the Rostock University Medical Center and University Hospital Schleswig-Holstein in Lübeck. In all experiments, both female and male mice were used. Homozygous C57BL/6N-Bach2^tm1b(EUCOMM)Wtsi^/leg mice (subsequently termed Bach2KO mice) were compared to wild type animals (termed Bach2WT mice) of the same strain. The mice were maintained under specific pathogen-free conditions with a 12-h light/dark cycle. They had access to water and rodent chow ad libitum. Animal experiments were conducted according to the European Community rules for animal care and performed by certified personnel. The animal study protocol was approved by the respective governmental administrations, Landesamt für Landwirtschaft, Lebensmittelsicherheit und Fischerei Mecklenburg-Vorpommern (protocol code 7221.3-1-015/22; date of approval: 22.07.2022) and the Ministry for Energy, Agriculture, Environment, and Rural Areas Schleswig-Holstein (protocol codes 60/7/22 and 59/7/22; date of approval: 12.10.2022).

### 2.2 Induction of AIP and treatment of mice

**Figure 1:**
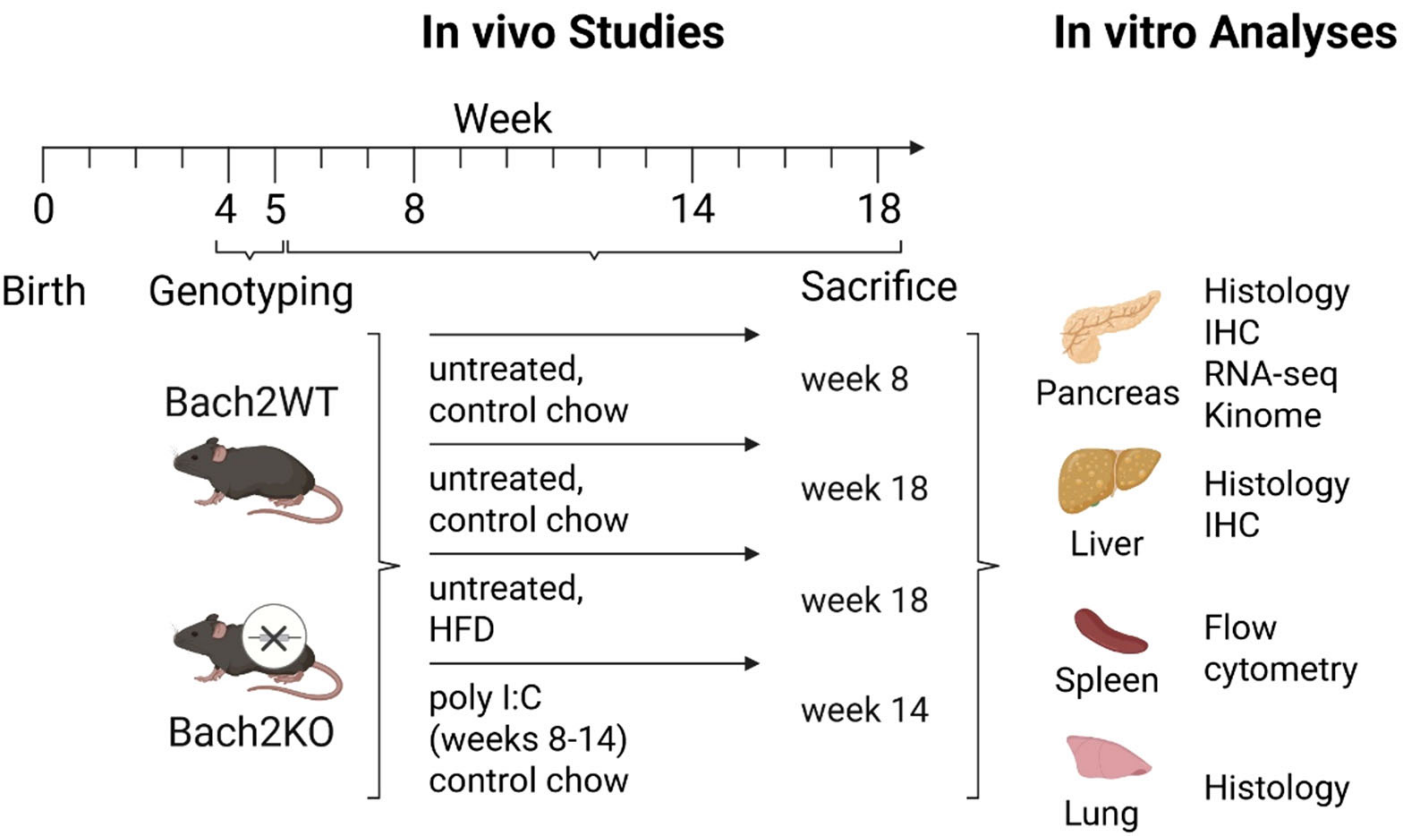
Overview of the experimental groups, workflow, and analyses. Each experimental group comprised 14–16 mice with largely equal sex distribution. For further details, please refer to the Materials and Methods. Bach2WT, *Bach2* wild type; Bach2KO, *Bach2* knockout; HFD, high-fat diet; poly I:C, polyinosinic:polycytidylic acid; IHC, immunohistochemistry.

Bach2WT and Bach2KO mice aged 4–5 weeks were randomly divided into experimental groups of 14–16 mice with a largely equal sex distribution. They were either left untreated (receiving control mouse chow), challenged with a high-fat “western” diet (HFD; ssniff Spezialdiäten GmbH, Soest, Germany, #S0587-E020), or received control mouse chow and intraperitoneal injections of poly I:C for 6 weeks (Miltenyi Biotec, Bergisch Gladbach, Germany; solvent: 0.9% NaCl; 5 mg/kg body weight, 3 injections per week) as described previously (4, 5). Further details are presented in **Figure 1**.

At the end of the investigation period, the mice were sacrificed using an overdose of ketamine/xylazine hydrochloride, followed by cervical dislocation. Blood samples, pancreas, spleen, and tissues were harvested and stored under appropriate conditions (mentioned below) until they were assayed.

### 2.3 Histology and immunohistochemistry

Formalin-fixed paraffin-embedded sections of pancreatic, liver, and lung tissues (4 µm thick) were stained with hematoxylin and eosin (H & E). After deparaffinization, the sections were additionally stained with antibodies against CD3 (BIOZOL Diagnostica, Hamburg, Germany; #DIA-303), CD138 (BioLegend, San Diego, CA, USA; #142502), CD68 (Cell Signaling Technology, Danvers, MA, USA; #97778T), or CD163 (Fisher Scientific, Schwerte, Germany; #30125032) using the ImmPRESS – alkaline phosphatase detection system (Vector Laboratories, Burlingame, CA, USA). The slides were counterstained with Mayer’s hemalum solution, dehydrated by incubating briefly in ethanol and xylene, and embedded in Pertex (MEDITE, Burgdorf, Germany).

Based on H & E and CD3 staining, the severity of pancreatic lesions was scored on a semi-quantitative scale from 0 to 4, as described previously for AIP in MRL/Mp mice (13, 35). Stage 0 (healthy pancreas) is characterized by the lack of inflammatory cells in the pancreatic parenchyma and the absence of any alteration in pancreatic morphology. Stage 1 was assigned to the pancreas with minimal focal infiltration of the periductal tissue with mononuclear cells but without parenchymal destruction. The presence of larger periductal foci of mononuclear cells and small areas of parenchymal destruction is characteristic of stage 2. Severe and multifocal periductal inflammation, together with more extensive parenchymal destruction and fibrosis, was assigned to stage 3. AIP stage 4, characterized by extensive destruction of the acini and severe fibrosis, was not observed in this study.

For detecting collagen in the liver tissue, Sirius Red staining was performed as described previously (36). To evaluate fatty liver disease, we used the histological scoring system established by Kleiner et al. (37) with some simplifications as listed in **Table 1**.

**Table 1:** Assessment of liver pathology.

Item	Definition	Score
Steatosis (Grade)	Low- to medium-power evaluation of parenchymal involvement by steatosis	0: less than 5% 1: 5-33% 2: 33-66% 3: more than 66%
Microvesicular steatosis	Contiguous patches	0: less than 5% 1: 5-33%
Inflammation	Overall assessment of inflammatory foci	0: No foci 1: less than 2 foci per 200x field 2: 2–4 foci per 200x field 3: more than 4 foci per 200x field
Liver cell injury	Hepatocellular ballooning	0: none 1: few balloon cells 2: many cells/prominent ballooning
Other findings	Mallory’s hyaline (visible on routine stains)	0: none to rare 1: many

All histological samples were evaluated in a blinded manner using light microscopy, and at least three tissue sections per sample were assessed.

### 2.4 Flow cytometry

Flow cytometry was performed as described previously (16). Briefly, single-cell suspensions from spleens were resuspended in freezing medium (10% dimethyl sulfoxide in fetal calf serum) and stored in liquid nitrogen until further use. After thawing, 0.5-1 × 10^6^ viable cells per sample were stained with the Zombie NIR™ fixable viability kit (BioLegend) and incubated with True-Stain monocyte blocker (BioLegend) and mouse FcR blocking reagent (Miltenyi Biotec) according to the instructions of the manufacturers. Next, the cells were incubated with the following fluorochrome-conjugated antibodies (all from Miltenyi Biotec) for 20 min at 4 °C: anti-CD45:PerCP, anti-CD44:PE-Vio770, anti-CD69:APC, anti-CD8a:VioBright FITC, anti-CD19:VioBright R720, and anti-CD62L:VioBlue. Prior to intracellular staining, the cells were permeabilized using 1× True-Nuclear Perm buffer (BioLegend). Subsequently, anti-CD3e:Brilliant Violet 605 and anti-CD4:Brilliant Violet 510 (both from BioLegend) were added and the cells incubated for 30 min at room temperature. For flow cytometric measurement, 100,000 cells per sample were analyzed using Cytek™Aurora (Cytek Biosciences, Amsterdam, The Netherlands) and data were evaluated using the FlowJo software (v10.10.0, Tree Star Inc., San Carlos, CA, USA). After exclusion of dead cells and doublets, CD45^+^ cells were differentiated as follows: CD3 was plotted against CD19, and CD19^+^CD3^-^ and CD3^+^CD19^-^ cells were recorded as B and T lymphocytes, respectively. From the CD3^+^CD19^-^ population, CD4^+^ and CD8^+^ cells were selected. CD4^+^ T lymphocytes were evaluated by gating the CD4^+^ T cells against CD69 and CD62L. CD8^+^ T lymphocytes were used to plot the CD8^+^ T cells against CD44, CD62L, and CD69.

### 2.5 RNA isolation and expression analysis

Total RNA from the stored spleen cells (see section 2.4) was isolated using the NucleoSpin RNA Plus XS assay (Macherey-Nagel, Düren, Germany). RNA was reverse transcribed into cDNA using TaqMan reverse transcription reagents (Thermo Fisher Scientific, Waltham, MA, USA), 250 ng RNA per sample, and random priming. For quantification of target cDNA levels using real-time PCR, a qPCR MasterMix from Eurogentec (Seraing, Liège, Belgium) and the following mouse-specific TaqMan^TM^ gene expression assays with fluorescently labeled MGB probes (Thermo Fisher Scientific) were used: Mm00477784_m (nuclear factor erythroid 2 related factor 2, *Nrf2*), Mm00516005_m (heme oxygenase 1, *Hmox1/Ho-1*), and Mm99999915_g1 (glyceraldehyde-3-phosphate dehydrogenase, *Gapdh*, house-keeping gene control). Polymerase chain reaction (PCR) was performed using the ViiA 7 sequence detection system (Thermo Fisher Scientific). The conditions were as follows: 95 °C for 5 min, followed by 40 cycles of 15 s at 95°C/1 min at 60°C. The relative amount of target mRNA in Bach2WT and Bach2KO cells was expressed as 2^−(Ct)^, where Ct = Ct_target mRNA_ - Ct_Gapdh_.

For RNA sequencing (RNA-seq), total RNA was isolated from homogenized pancreatic tissue using the guanidinium thiocyanate/phenol method, as described by Sparmann et al. (38). After quantification using spectrophotometry (NanoDrop 1000; Thermo Fisher Scientific), RNA integrity was validated using an Agilent Bioanalyzer 2100 with an RNA Nanochip kit (Agilent Technologies, Waldbronn, Germany). RNA-seq analysis was performed by Novogene (Munich, Germany). After mRNA library preparation, the samples were sequenced using a NovaSeq X Plus system (Illumina, Cambridge, UK) and a paired-end 150 bp (PE150) reading strategy. The number of clean reads was in the range of 1−2 × 10^9^ per sample.

### 2.6 Immunoblotting

Protein extracts from isolated spleen cells were separated using sodium dodecyl sulfate-polyacrylamide gel electrophoresis and the proteins were blotted onto polyvinylidene fluoride membranes (Merck Millipore, Darmstadt, Germany). Blots were incubated with antibodies against HO-1 (Fisher Scientific; #16877253) or the house-keeping protein, β-actin (Cell Signaling Technology; #4970) overnight at 4 °C and developed using LI-COR reagents for an Odyssey^®^ infrared imaging system (LI-COR Biosciences, Lincoln, Nebraska USA) as described previously (39). Signal intensities were quantified using the Odyssey^®^ software version 3.0 and the raw data was processed as described in the figure legends.

### 2.7 Multiplex kinase activity profiling by PamGene™

Kinase activity profiles were determined using the PamChip^®^ 4 protein tyrosine kinase (PTK) peptide microarray system from PamGene™ International B.V. (BJ’s-Hertogenbosch, The Netherlands) as described previously (40, 41). Four 60 μm-thick frozen pancreatic tissue slices were cut and lysed using M-PER (mammalian protein extraction reagent; Thermo Fisher Scientific) containing 1% (v/v) Halt phosphatase inhibitor cocktail (Thermo Fischer Scientific) and 2% (v/v) Halt protease inhibitor cocktail (Thermo Fisher Scientific). After centrifugation (15 min, 4°C, 10,000 × g), the supernatants were snap-frozen and stored at -80°C. Protein concentration was determined using a bicinchoninic acid assay kit (Thermo Fischer Scientific) according to the manufacturer’s instructions. The serine-threonine kinase (STK) and PTK microarray assays were performed according to the manufacturer’s instructions. A kinase was considered modulated (either activated or inhibited) if it had a mean specificity score of 1 and a significance score of 0.5 (p = 0.32). Human UniProt IDs were translated into mouse UniProt IDs based on high conservation levels and homology of kinases (42). Hence, the data obtained indicated differences in kinase activities when comparing Bach2KO to Bach2WT. The mean kinase statistics was used for further analysis, the proteome was visualized using the Proteomaps database (43), and kinome trees were created using the Coral (44) and STRING databases (45).

### 2.8 Statistics

The sample size for the mouse study was calculated using SigmaPlot 13.0 (Grafiti LLC, Palo Alto, CA, USA). The primary endpoint was pancreatic histopathology, as assessed by scoring (see Section 2.3). Considering an α-error of 0.05 and power of 85%, a group size of 16 mice was required to detect a difference of 0.6 units in histological score (with an expected SD of 0.4). Statistical analyses of RNA-seq data were performed by Novogene. Other data were analyzed using GraphPad Prism (GraphPad Software, version 10.4.1, San Diego, CA, USA). The normal distribution of the data was tested using the Kolmogorov-Smirnov and Shapiro-Wilk tests. The sample sizes and statistical tests used for the data analysis are provided in the figure legends. Statistical significance was set at p < 0.05.

## 3 Results

### 3.1 Phenotypic features of Bach2KO mice

**Figure 2:**
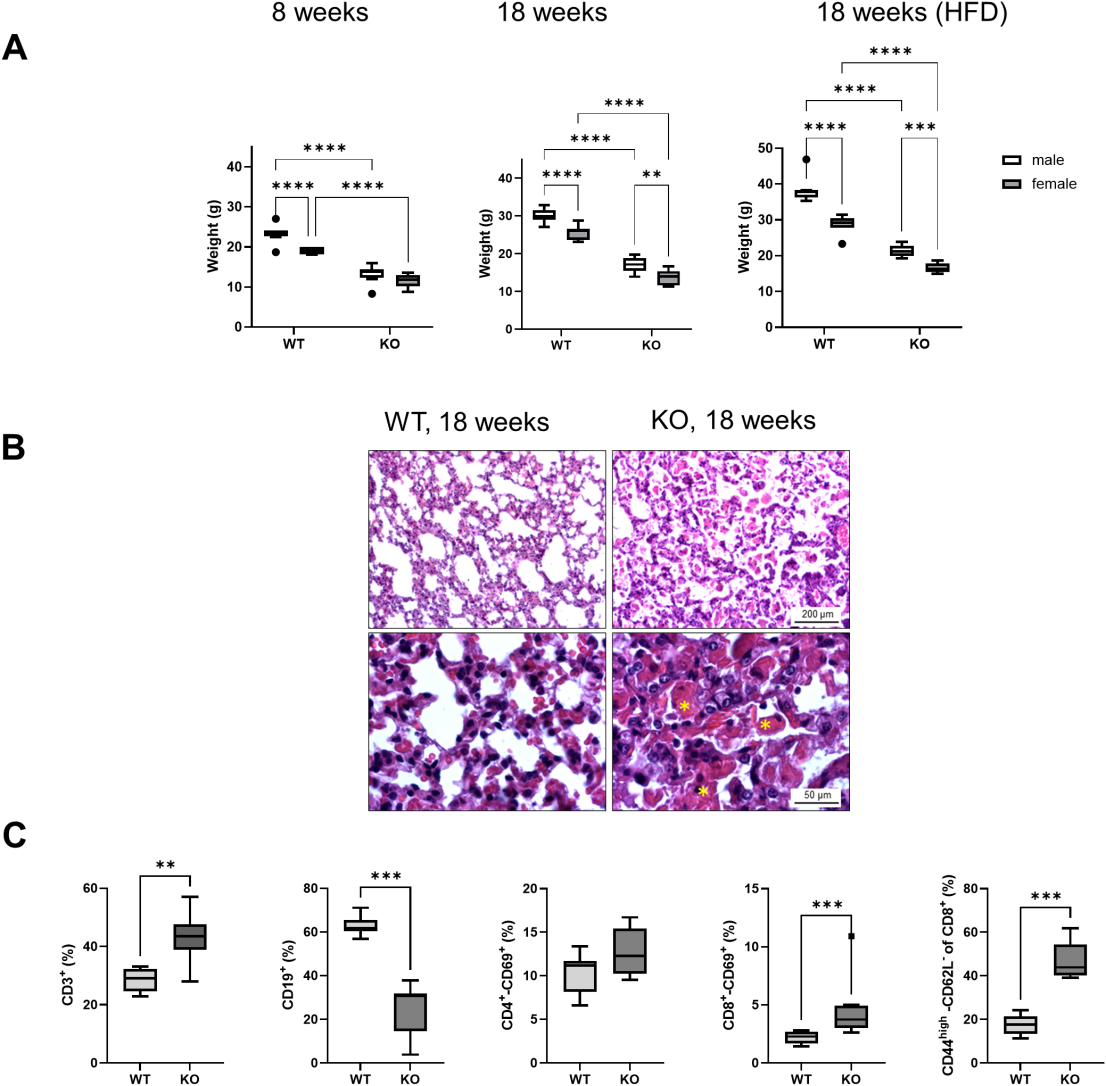
Phenotypic features of Bach2KO mice. **(A)** Bach2WT and Bach2KO mice of the experimental groups, 8 weeks untreated, 18 weeks untreated, and HFD (see **Figure 1**) were weighed on the last day of the experiment. Data are shown as box plots (Tukey) with median (horizontal line). Number of mice: 8 males and 8 females per experimental group. **p < 0.01, ***p < 0.001, and ****p < 0.0001 (two-way ANOVA with uncorrected Fisher’s least significant difference test). **(B)** Sections of lung tissue were stained with hematoxylin and eosin. Asterisks indicate deposits of eosinophilic materials. Original magnifications: 100× and 400×. The findings are representative of 16 mice (8 males and 8 females) of the indicated experimental groups. **(C)** Relative frequencies of lymphocyte subtypes in the spleen. Cells isolated from the spleens of 18-week-old untreated Bach2WT and Bach2KO mice (n = 4 males and 4 females per experimental group) were subjected to flow cytometry using labeled antibodies against CD antigens, and the relative frequencies of the indicated lymphocyte subpopulations were determined. Data are presented as box plots (Tukey) with median (horizontal line). **p < 0.01 and ***p < 0.001 (Mann-Whitney test). Bach2WT, *Bach2* wild type; Bach2KO, *Bach2* knockout; HFD, high-fat diet.

Consistent with the results of previous studies (46), Bach2KO mice with a global knockout of the *Bach2* gene were smaller and lighter than their Bach2WT age- and sex-mates. Significant weight differences were observed at 8 and 18 weeks of age in both the standard mouse diet-fed and HFD-fed mice. As expected, male mice in all groups were heavier than female mice, and mice fed HFD gained more weight than those fed the standard diet (**Figure 2A**). Bach2KO mice develop eosinophilic crystalline pneumonia in early adulthood, which is a fatal lung disease (47). For reasons of animal welfare, we euthanized all Bach2KO mice at a maximum age of 18 weeks when no severe respiratory distress was detected. In agreement with the findings of Kim et al. (47), histological analysis of the lungs of 18-week-old Bach2KO mice showed a loss of alveolar spaces caused by exudates, interstitial infiltrates, and deposition of eosinophilic materials, whereas no such pathologies were observed in Bach2WT mice (**Figure 2B**).

Flow cytometric analysis of spleen cells from 18-week-old Bach2KO mice revealed several immunological abnormalities. Compared with Bach2WT animals, there was a predominance of CD3^+^ T lymphocytes over CD19^+^ B cells. Furthermore, significantly more CD3^+^-CD8^+^ (but not CD3^+^-CD4^+^) cells from Bach2KO mice than from Bach2WT animals expressed the early activation marker, CD69, and significantly higher numbers of CD8^+^-CD44^high^-CD62L^-^ memory effector T cells were observed (**Figure 2C**). The gene expression of two key components of the Bach2 reaction network, *HO-1* and *Nrf2*, did not differ significantly in the spleen cells of Bach2WT and Bach2KO cells. For HO-1, this finding was confirmed at the protein level (**Supplementary Figure S1**). Regarding the known repressive effect of Bach2 on *HO-1* (48), the significance threshold was only narrowly missed (p = 0.054).

### 3.2 Bach2KO mice develop pancreatic lesions typical of AIP

**Figure 3:**
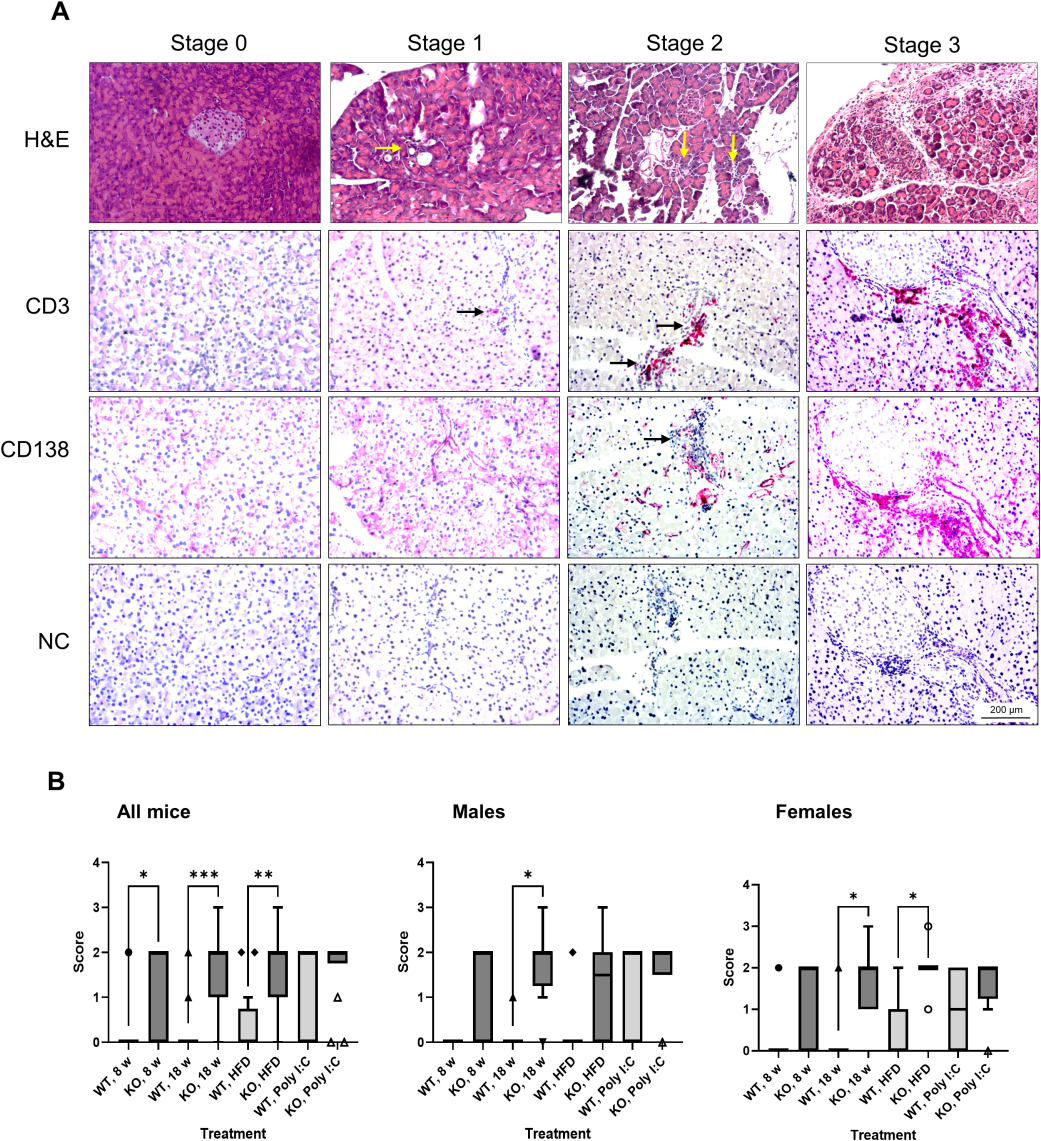
Evaluation of pancreatic histopathology. Bach2WT and Bach2KO mice were either left untreated (8 weeks and 18 weeks groups), fed with HFD, or administered injections of poly I:C as described in the Materials and Methods. **(A)** Upper row (H & E staining). The photographs display representative examples of pancreatic lesions for the AIP stages 0 (healthy pancreas), stage 1 (minimal focal infiltrates), stage 2 (moderate focal lymphocytic infiltration and locally restricted destruction of acinar tissue), and stage 3 (multifocal inflammation, more extensive destruction of acinar tissue, and fibrosis). The arrows point to small infiltrates of inflammatory cells. Lower rows (immunohistochemical analyses). The samples were immunostained for CD3 and CD138 as indicated, while negative controls (NC) were treated with the secondary antibody only (which was identical for CD3 and CD138 staining). Positively stained cells (indicated by arrows) appear red/magenta. Representative microscopic images are shown for each stage of AIP. Original magnifications: 100× and 200×. **(B)** The pancreatic lesions were evaluated by applying the scoring system as described above and in the Materials and Methods. Data are shown as box plots (Tukey) with median (horizontal line). Number of mice: 6–8 males and 7–8 females per experimental group; *p < 0.05, **p < 0.01, and ***p < 0.001 (Kruskal-Wallis test, *post-hoc* analysis; Dunn’s multiple comparison test). H & E, hematoxylin and eosin; NC, negative control; Bach2WT, *Bach2* wild type; Bach2KO, *Bach2* knockout; HFD, high-fat diet; poly I:C, polyinosinic:polycytidylic acid.

To determine the influence of the *Bach2* gene knockout on pancreatic histology, we examined mice aged 8 and 18 weeks, the latter with and without HFD, and animals that had been treated for 6 weeks with poly I:C as a potential trigger of an autoimmune disease. The histopathological changes were grouped by stage and are shown in **Figure 3A**. These findings are typical of AIP stages 0–3 described in the Materials and Methods. Although few or no inflammatory cells were present in stages 0 and 1, larger infiltrates and smaller lesions were observed in stage 2. In stage 3 samples, extensive infiltration, destruction, and fibrosis were detected. The inflammatory foci were largely composed of CD3^+^ T cells and macrophages expressing the M1 marker, CD68, or the M2 marker, CD163 (**Supplementary Figure S2A**); however, CD138^+^ plasma cells were also observed (**Figure 3A**).

In untreated 8- and 18-week-old Bach2WT mice, histological alterations were rare and stage 0 was a typical finding (**Figure 3B**, left panel). In the corresponding Bach2KO groups, significantly higher scores were observed. HFD feeding and poly I:C injections were not associated with significantly higher scores in either Bach2WT or Bach2KO mice than in unchallenged mice of the same genotype. However, the significant difference between the genotypes was lost after poly I:C injections, suggesting some effect of the trigger substance in the Bach2WT animals. Separate analyses of males and females showed similar patterns without fundamental differences (**Figure 3B**, middle and right panels). Taken together, these histological findings suggest that Bach2KO mice are prone to AIP.

**Figure 4:**
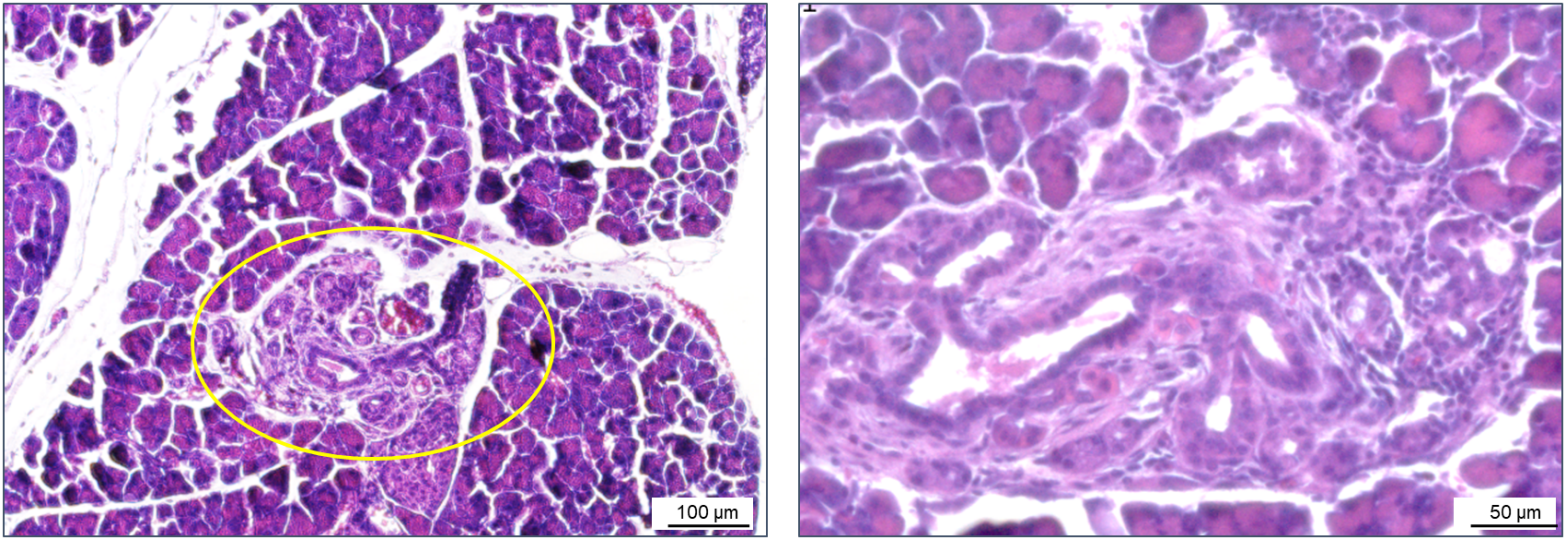
ADM in a Bach2KO mouse. Sections of pancreatic tissue from a Bach2KO mouse (18-week-old, untreated) were stained with hematoxylin and eosin. The area of suspected ADM is encircled; original magnifications: 100× and 200×. ADM, acinar-to-ductal metaplasia; Bach2KO, *Bach2* knockout.

In exceptional cases, areas of suspected acinar-to-ductal metaplasia (ADM) were observed in the pancreatic tissues of Bach2KO (but not in Bach2WT) mice (**Figure 4**). ADM is considered a reversible process that enables the regeneration of pancreatic acini. However, persistent inflammation can progress to pancreatic intraepithelial neoplasia, a common precursor of pancreatic ductal adenocarcinoma (49).

### 3.3 RNA-sequencing and kinome assays suggest activation of autoimmune processes in pancreatic tissue of Bach2KO mice

**Figure 5:**
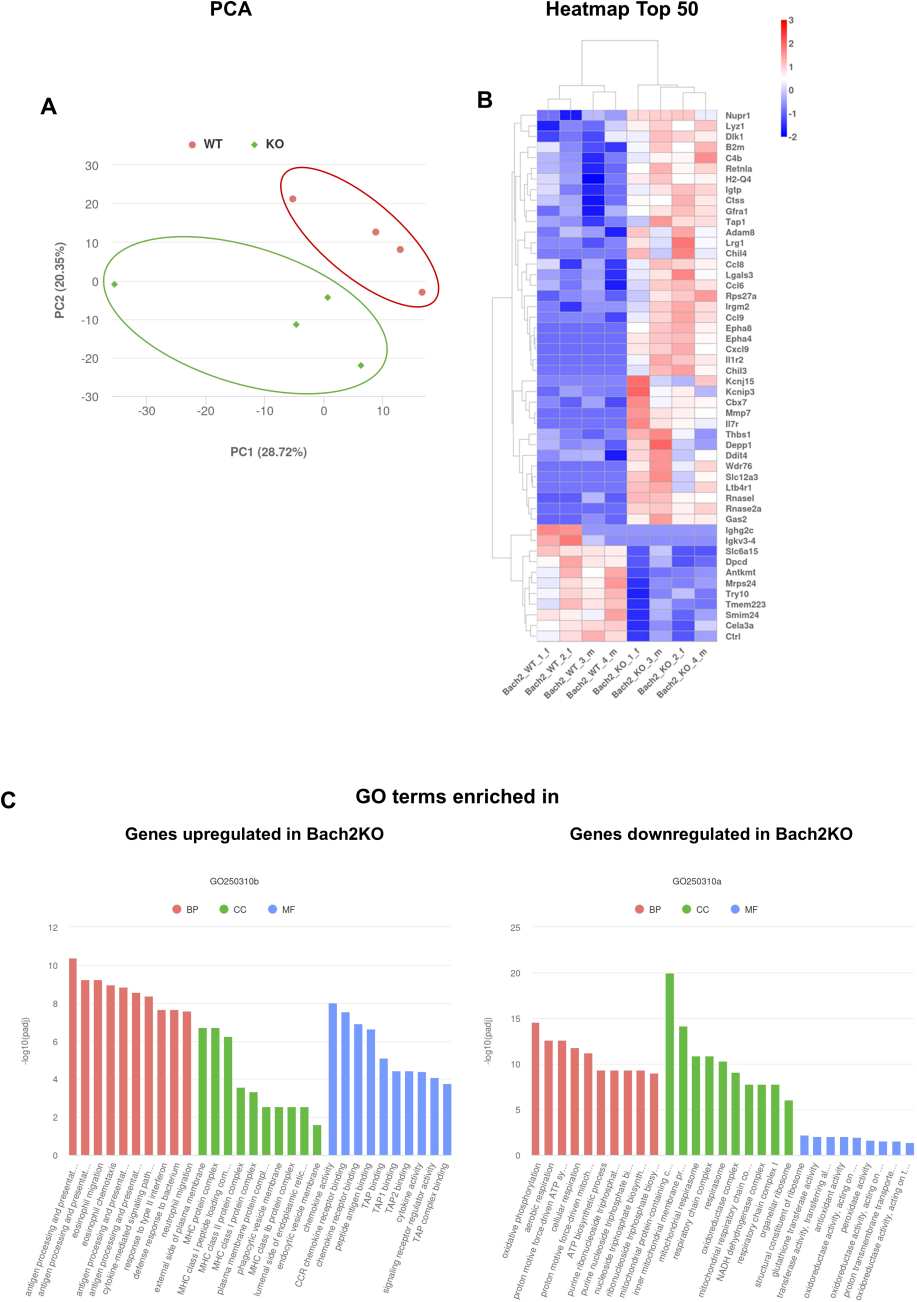
RNA-seq analyses of pancreatic samples derived from 18-week-old untreated mice. The diagrams are based on the analysis of two female and two male *Bach2* wild type mice (termed Bach2_WT^_1_f^, 2_f, 3_m, and 4_m), and two female and two male *Bach2* knockout mice (termed Bach2_KO^_1_f^, 2_f, 3_m, and 4_m). **(A)** Principal component analysis (PCA) of gene expression profiles (with manually encircled clusters). **(B)** Clustering heatmap of the top 50 differentially expressed genes (up- or down-regulated) from the comparison of Bach2WT versus Bach2KO mice, based on the adjusted p-values. **(C)** GO term enrichment analysis for transcripts enriched in Bach2WT and Bach2KO mice, based on the adjusted p-values. The adjustment for multiple hypothesis testing was performed using the Benjamini-Hochberg false discovery rate method. Bach2WT, *Bach2* wild type; Bach2KO, *Bach2* knockout; GO, Gene Ontology; BP, Biological Process; CC, Cellular Component; MF, Molecular Function. A list of the top 200 genes, along with log2FoldChanges and the corresponding adjusted p-values for all comparisons, is provided in **Supplementary Table S1**. The details of the GO terms are listed in **Supplementary Table S2**.

To gain insight into pancreatic gene expression profiles, pancreatic tissues derived from 18-week-old untreated mice were subjected to RNA-seq. Principal component analysis (PCA) revealed that Bach2WT and Bach2KO mice formed separate clusters (**Figure 5A**). A clustering heatmap of differentially expressed genes (**Figure 5B**) and Gene Ontology (GO) term enrichment analysis (**Figure 5C**) showed that the genes and terms that were upregulated in Bach2KO mice were predominantly associated with activated immune responses, such as antigen processing and presentation, migration, chemotaxis, and cytokine/chemokine action. For genes downregulated in Bach2KO mice, enrichment of GO terms linked to energy production via aerobic metabolism was observed.

**Figure 6:**
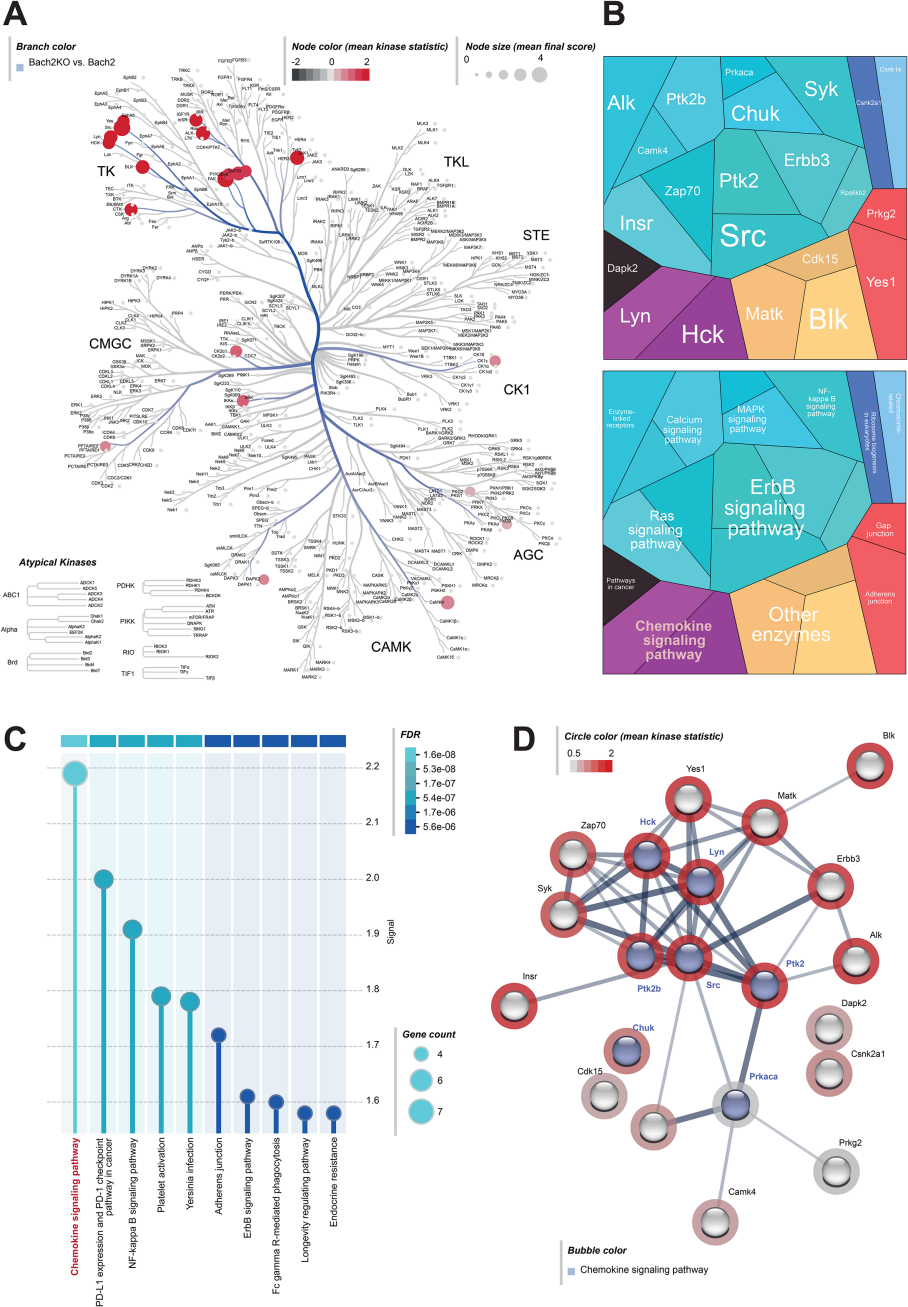
Kinome analysis of pancreatic samples derived from 18-week-old untreated mice. Pancreatic tissues of untreated Bach2WT and Bach2KO mice were analyzed for kinase pathways using chip-based kinome activity assays. **(A)** Kinome tree of all up-regulated PTKs and STKs in Bach2KO mice. Node size: mean final score; node color: mean kinase statistic. **(B)** Two different hierarchy levels of proteomaps and **(C)** KEGG pathway analysis showing upregulated kinases and signaling pathways in Bach2KO compared to that in Bach2WT mice. **(D)** STRING pathway analysis of the relevant activated kinases in Bach2KO mice (red circle: mean kinase statistic), indicating the kinases involved in the “chemokine signaling pathway”, (highlighted in blue). Data is based on three mice (males only) per group. Bach2WT, *Bach2* wild type; Bach2KO, *Bach2* knockout; PTK, protein tyrosine kinase; STK, serine-threonine kinase; KEGG, Kyoto Encyclopedia of Genes and Genomes.

To further investigate the changes in signaling pathways in the pancreatic tissues of Bach2WT and Bach2KO mice, we performed a chip-based kinome analysis. Interestingly, many PTKs involved in T cell activation, such as the SRC family members, SRC, LYN, YES, ZAP-70, FAK1/2, and Syk (50, 51), were activated in Bach2KO mice (**Figures 6A, B**). In agreement with this, the most prominent Kyoto Encyclopedia of Genes and Genomes (KEGG) pathways found in the pancreatic tissue of BachKO mice were related to chemokines (**Figures 6C, D**) and PD−1/PDL1 signaling (**Figure 6C**).

Overall, the RNA-seq and kinome data were consistent with the activation of autoimmune processes in the pancreas of Bach2KO mice.

### 3.4 Bach2KO mice are protected from HFD-induced fatty liver disease

**Figure 7:**
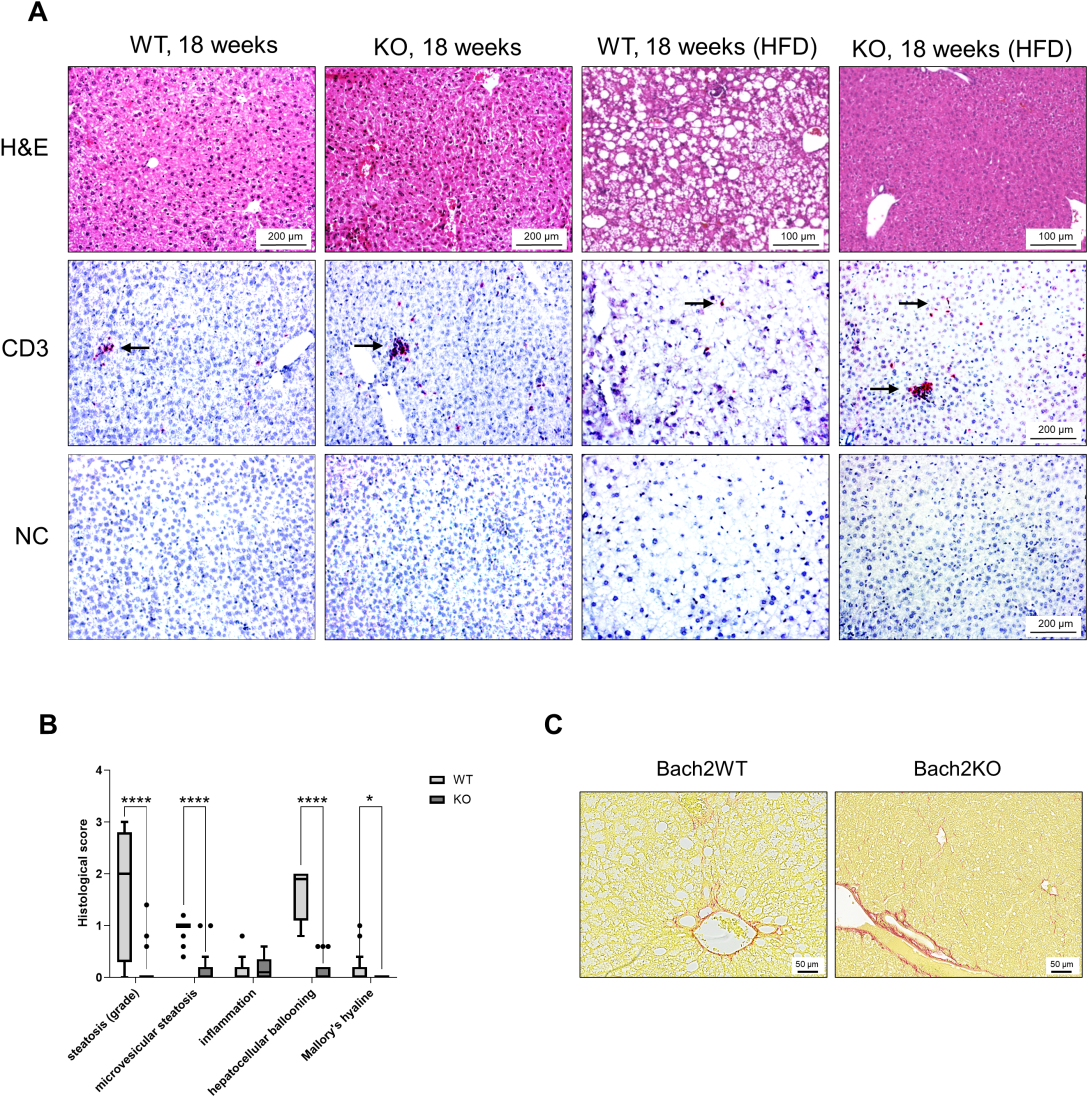
Evaluation of liver histopathology. Bach2WT and Bach2KO mice were either left untreated (18 weeks groups) or fed with HFD as described in the Materials and Methods. Each experimental group consisted of 8 males and 8 females. **(A)** Upper row: H & E staining; lower rows: immunohistochemical analyses. The samples were immunostained for CD3 as indicated, while negative controls (NC) were treated with only the secondary antibody. Positively stained cells (indicated by arrows) appear red/magenta. Representative microscopic images are shown for each group. **(B)** Fatty liver disease of HFD-fed mice was assessed by applying a scoring system as described in the Materials and Methods. Data are shown as box plots (Tukey) with median (horizontal line). *p < 0.05 and ****p < 0.0001 (Mann-Whitney-test). **(C)** Liver tissues of HFD-fed mice were subjected to staining with Sirius Red for collagen visualization. The findings are typical for the respective experimental groups. Original magnifications: 100× and 200×. H & E, hematoxylin and eosin; NC, negative control, Bach2WT, *Bach2* wild type; Bach2KO, *Bach2* knockout; HFD, high-fat diet.

As a quality control for HFD, histological examinations of mice livers were performed (**Figure 7**), aiming to document fatty liver disease. The livers of untreated 18-week-old Bach2WT and Bach2KO mice did not show gross abnormalities upon H & E staining. As expected, the livers of HFD-fed Bach2WT mice showed severe steatosis and hepatocyte ballooning, consistent with fatty liver disease, whereas inflammatory cell infiltration was absent. Unexpectedly, HFD-fed Bach2KO mice did not develop hepatic steatosis and there was no damage to the hepatocytes (**Figure 7A**, upper row). In both genotypes, low number of CD3^+^ T cells were observed, either as single cells or small clusters. CD3^+^ T cells were particularly rare in the liver tissue of HFD-fed Bach2WT mice (**Figure 7A**, lower rows), whereas CD68^+^ and CD163^+^ macrophages were present at similar densities in both Bach2WT and Bach2KO mice (**Supplementary Figure S2B**). A histological scoring system (**Table 1**) was used to confirm the findings. Parenchymal involvement indicated by steatosis, microvesicular steatosis, hepatocyte ballooning, and the presence of Mallory’s hyaline was significantly higher in Bach2WT than in Bach2KO mice (**Figure 7B**). Separate analysis of male and female mice revealed that general liver steatosis was largely restricted to male Bach2WT mice, whereas the differences between the genotypes regarding microvesicular steatosis and hepatocyte ballooning were independent of sex (**Supplementary Figure S3**). Collagen staining showed that neither the HFD-fed Bach2WT nor Bach2KO mice developed pronounced fibrosis (**Figure 7C**). Taken together, these data suggest that *Bach2*-deficient mice are protected from HFD-induced fatty liver disease.

## 4 Discussion

In previous genome-wide association studies, we have identified *Bach2* as a potential risk gene for murine AIP (4). Here, we used Bach2KO mice to study their susceptibility to AIP and gain additional mechanistic insights into the pathogenesis of the disease. The reproducibility of preclinical animal research has been shown to improve with heterogeneity of the study samples (52). We considered this important aspect by performing all the studies in parallel at two sites with independent mouse colonies and in animals of both sexes. As AIP developed spontaneously in Bach2KO mice, we concluded that *Bach2* played a pathogenic role in experimental AIP.

Although this is the first study to directly implicate *Bach2* in the pathogenesis of AIP, genetic variations within the human *BACH2* locus have generally been associated with CP (30). This supports the concept that *Bach2*-dependent autoimmunological processes are involved not only in AIP but also in the progression of more common forms of CP. The transcriptional repressor, Bach2, is predominantly expressed in B and T lymphocytes, where it regulates terminal differentiation and maturation. Consistent with the established functions of Bach2 in immune cells, we observed increased infiltration of exocrine pancreatic tissue by CD3^+^ T cells, CD138^+^ plasma cells, and macrophages. Although this was accompanied by incipient destruction of the pancreatic tissue and occasional fibrosis, the overall histological changes were mild compared to those in AIP-prone MRL/MpJ mice. This may be because additional genetic traits present in the latter strain may be absent in the Bach2KO mice that has a C57BL/6 background.

In this study, we investigated the interplay between genetic risks and environmental factors by feeding subgroups of mice a HFD or injecting them with poly I:C. Previously, we and others have shown poly I:C to be a potent trigger of AIP in mice (14, 31). HFD tended to be associated with more severe disease stages, whereas a low-calorie diet showed a protective effect (4). Importantly, these earlier studies were conducted in mice that were prone to spontaneously developing the disease (MRL/MpJ strain and outbred mice with the genetic traits of MRL/MpJ mice). In contrast, the Bach2 mice used in this study had a C57BL/6N background, which does not predispose them to autoimmune diseases at young age. The results of the current study, specifically the histological findings, RNA-seq data, and results of the PamGene multiplex kinase activity assays, showed that Bach2KO mice are prone to spontaneous AIP. In contrast, Bach2WT mice developed the disease neither spontaneously nor after HFD. Statistically significant differences between the genotypes were no longer detectable after administration of the strong trigger of poly I:C. The pancreatic phenotype of Bach2KO mice correlated with the presence of more early activated and memory effector CD8^+^ T cells in the spleen, implicating disturbed immune homeostasis with dysregulated activation of adaptive immune system cells in the pathogenesis of the disease.

In addition, our investigations yielded a second and unexpected result: we found that only Bach2WT mice fed a HFD developed fatty liver disease. Bach2KO mice were protected and showed largely normal liver histology after approximately 3 months of HFD feeding. In humans, metabolic dysfunction-associated steatotic liver disease is a serious public health issue estimated to affect more than one-third of adults worldwide. MASLD is closely associated with insulin resistance, obesity, dyslipidemia, arterial hypertension, and genetic risk factors. Among the different phases of liver injury summarized under the term MASLD, isolated hepatic steatosis is the earliest and potentially reversible stage. However, steatosis can develop via metabolic dysfunction-associated steatohepatitis into a progressive necroinflammatory disease, with a significant risk of further progression to fibrosis, cirrhosis, and hepatocellular carcinoma (53, 54). The main features of the liver histopathology in HFD-fed Bach2WT mice were steatosis and hepatocyte ballooning, whereas no marked inflammation or fibrosis was observed, consistent with a relatively early stage of liver injury.

The finding that elimination of the inflammatory inhibitor, Bach2, exerts a protective effect in fatty liver disease appears to be initially intriguing. The results of a bioinformatics study by Li et al. (55) on microarray datasets from patients with MASLD are therefore all the more remarkable. The authors originally identified 18 immune-related differentially expressed genes. After further analyses, only two genes were upregulated in MASLD, and *BACH2* was one of the upregulated genes (in addition to *S100A9*).

However, the molecular and immunological mechanisms that underlie Bach2 action in HFD-induced fatty liver disease still need to be investigated. Notably, lower numbers of CD4^+^ T cells have been reported in the liver of patients (56), supporting the hypothesis that immune function is suppressed in MASLD (55). The elimination of the repressor effect of Bach2 in our mouse model could therefore lead to the reactivation of immune response, which in turn prevents the development of liver steatosis. However, the role of autoaggressive T cells (CD8^+^-CXCR6^+^) in the pathogenesis of MASLD should not be overlooked (57). The direct effects of *Bach2* knockout in resident liver cells may also play a role in preventing fatty liver disease, although the data regarding this is limited. Silencing of *Bach2* in the HepG2 cell line is associated with decreased lipid accumulation (55). It is noteworthy that *Bach2* deficiency was found to promote intestinal epithelial regeneration by accelerating DNA repair in intestinal stem cells (58). Future studies should investigate whether a similar mechanism leads to improved regeneration of hepatocytes from *Bach2*-deficient stem cells.

One limitation of the constitutive *Bach2* knockout model used in this study was the severe phenotype of the mice, which had to be euthanized after 18 weeks to minimize suffering from the previously characterized lung disease (46, 47). While this period was sufficient for the development of mild AIP and fatty liver disease caused by the HFD, longer follow-up periods are necessary to observe more advanced disease stages, such as liver fibrosis and cirrhosis. The short observation period also prevented further investigation of possible consequences of ADM in Bach2KO animals, which may increase the risk of developing precancerous lesions and pancreatic cancer. The use of conditional/cell-specific *Bach2* knockout models with less severe phenotypes is a logical next step, and it is expected to yield additional mechanistic insights.

**Figure 8:**
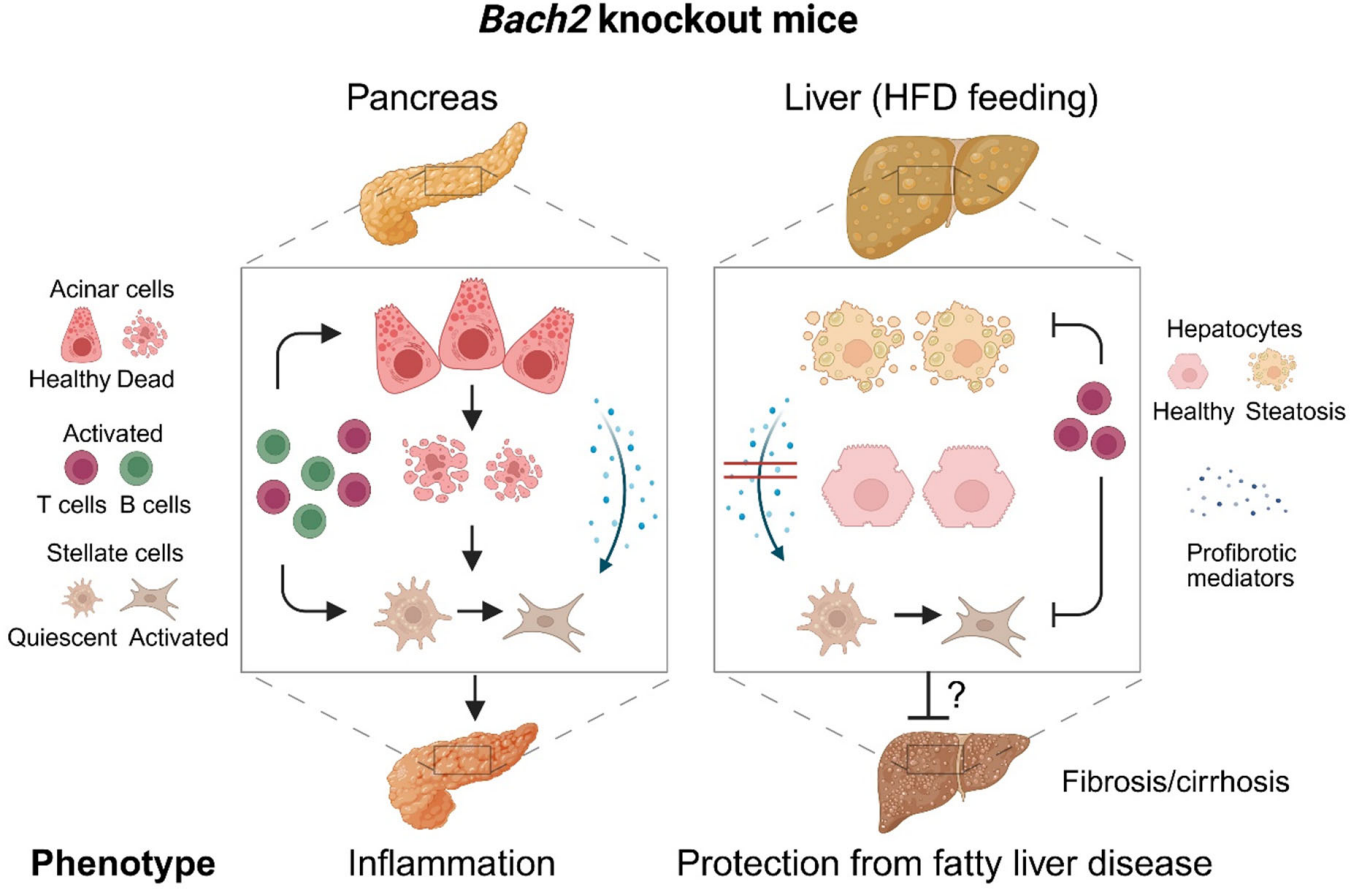
Effects of *Bach2* knockout in the pancreas and liver. Pancreas: While the pancreas of wild type mice was healthy, *Bach2* knockout mice developed mild autoimmune pancreatitis with infiltrates of T cells and plasma cells, an incipient destruction of pancreatic tissue, and some fibrosis. Pancreatic fibrosis, similar to hepatic fibrosis, is caused by the activation of stellate cells (59). Liver: In response to HFD-feeding, *Bach2* wild type mice developed a fatty liver disease, while the liver of *Bach2* knockout mice remained healthy. We hypothesize that T cells are involved in mediating the protective effect of the gene knockout, as they were present in higher numbers than in the liver tissue of HFD-fed wild type mice. Within the relatively short observation period (up to the 18th week of life), fatty liver disease did not progress to cirrhosis even in wild type mice. The persistence of the protective effect of the gene knockout should therefore be further investigated in follow-up studies, as should the molecular mechanisms of Bach2 action. HFD, high-fat diet. Created in BioRender. Jaster, R (2025). https://BioRender.com/ph4c91u.

Collectively, our results establish *Bach2* as a protective genetic factor in experimental AIP and provide the first evidence that *Bach2* potentially contributes to the pathogenesis of MASLD in mice. The key findings of this study are presented in **Figure 8**. Whether BACH2 is a suitable therapeutic target in patients with MASLD, as postulated (55), should be further investigated in preclinical studies.

## Data Availability

The complete RNA-seq data will be available in the Gene Expression Omnibus database (60) (GEO accession number: GSE296473). Further raw data supporting the conclusions of this article will be made available by the authors on request and without reservation.
