# Supplementary figures and images for: Bach2-deficient mice are prone to autoimmune pancreatitis but protected from high-fat diet-induced fatty liver disease
Source: Front Immunol. 2025 Oct 15;16:1639622. doi: 10.3389/fimmu.2025.1639622 (PMC12568605; doi:10.3389/fimmu.2025.1639622)

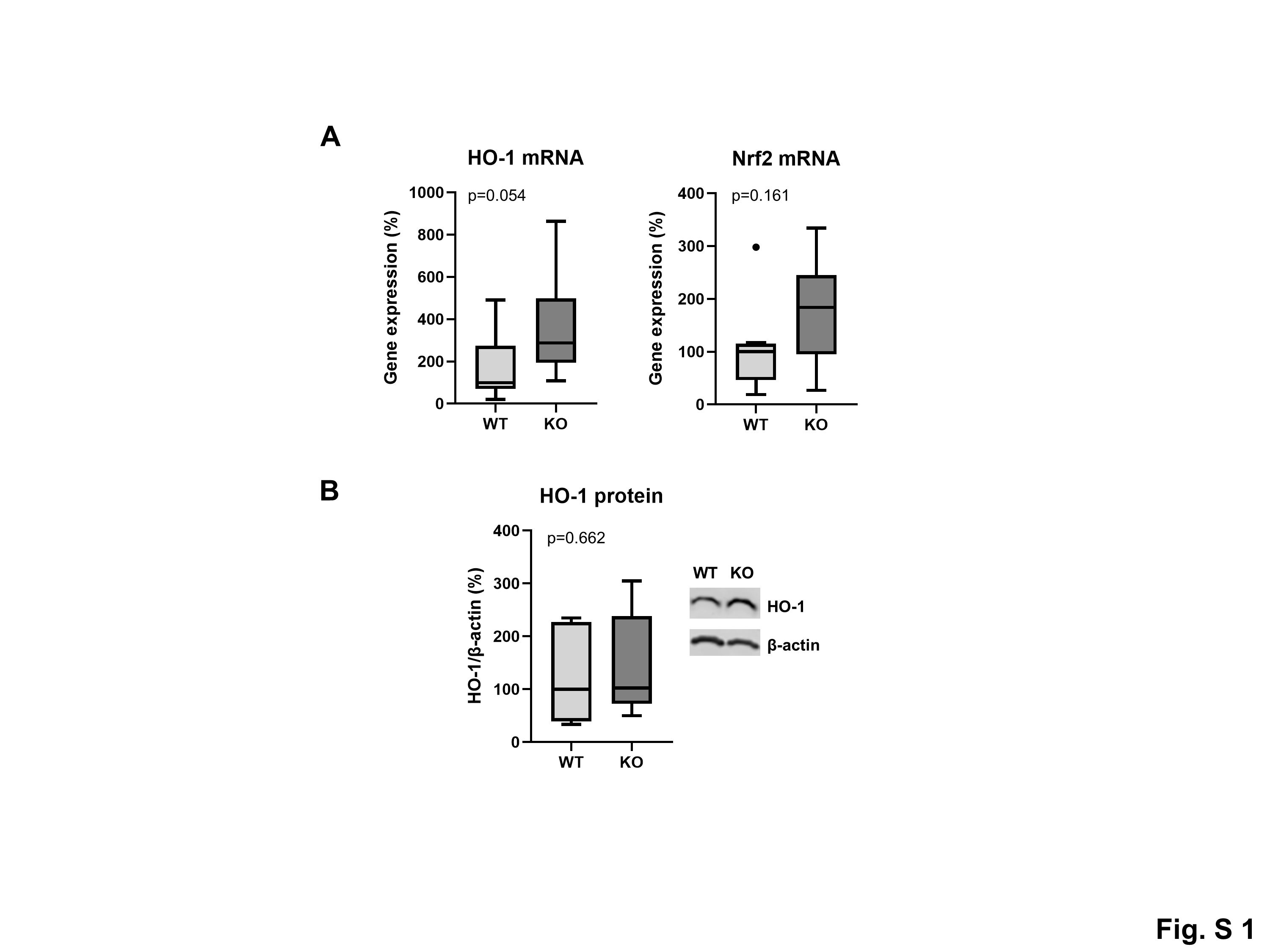

Supplement: Supplementary file 1 [file Image1.tif]

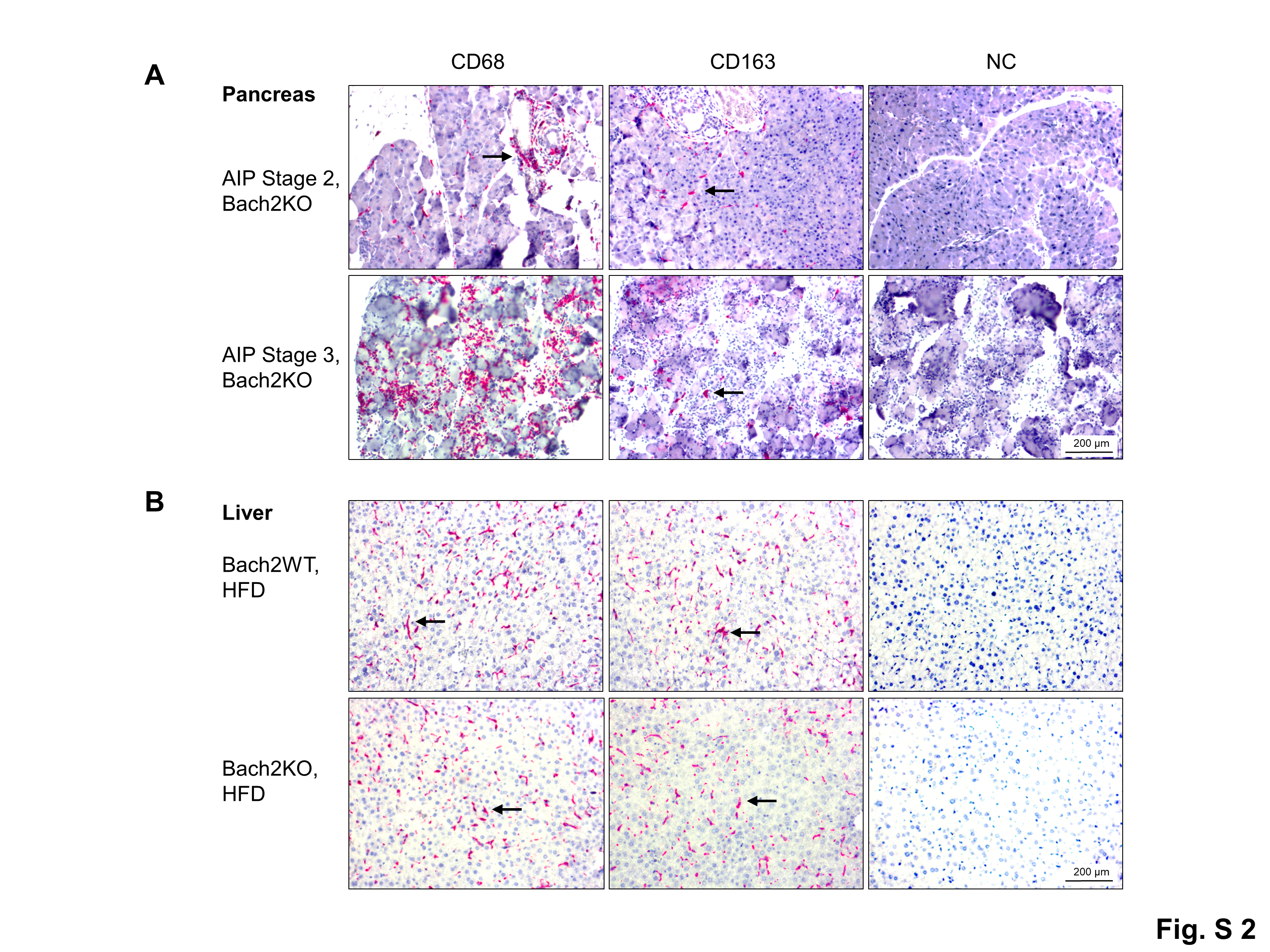

Supplement: Supplementary file 2 [file Image2.tif]

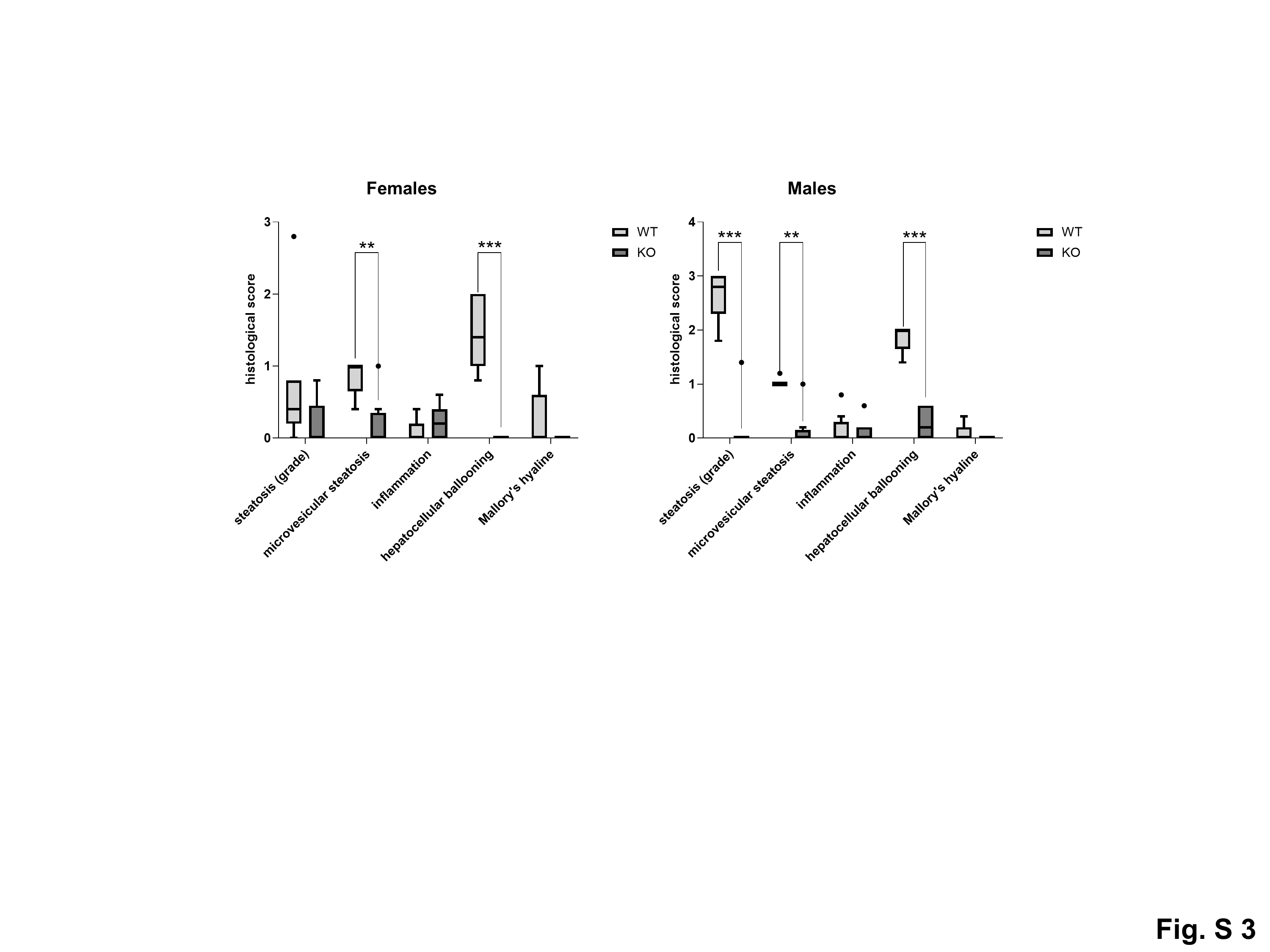

Supplement: Supplementary file 3 [file Image3.tif]
